# Editorial: Evolution and mechanisms of anti-malarial and insecticide resistance

**DOI:** 10.3389/fcimb.2023.1274741

**Published:** 2023-08-29

**Authors:** Linda E. Amoah, Eugenia Lo

**Affiliations:** ^1^ Department of Immunology, Noguchi Memorial Institute for Medical Research, University of Ghana, Accra, Ghana; ^2^ Department of Microbiology and Immunology, College of Medicine, Drexel University, Philadelphia, PA, United States

**Keywords:** drug resistance, antimalarial drugs, mechanisms, evolution, insecticide resistance

Malaria, a deadly disease caused by *Plasmodium* spp., continues to pose a significant threat to global health. This is further exacerbated by antimalarial drug and insecticide resistances. This is unfortunate, as chemical therapy and preventive medicine remain as our main defenses. This editorial highlight ongoing strategies to mitigate malaria, including the surveillance of drug and insecticide resistance and drug discovery.

Human migration is a major problem for malaria control and elimination efforts as infected people can disperse the parasites during their movement. Drug resistant malaria parasites, especially those resistant to the drug family artemisinin are also a threat to malaria control and elimination efforts. Zhao et al. examined the drug susceptibility profile of parasite isolates in Chinese travelers returning from Ghana with uncomplicated malaria. The parasite isolates were susceptible to artemisinin and the partner drugs of artemisinin-based combination therapies but markedly resistance to antifolate drugs. A low prevalence of chloroquine-resistant genes was consistent with the suspension of chloroquine therapy.

On the drug-innovation front, Burns et al. explored the antiparasitic activity of 22 azithromycin analogues against *P. falciparum* and *P. knowlesi* and identified 17 analogues with almost 40-fold activity relative to azithromycin. Metabolomic profiling of parasites treated with the most potent compound showed a build-up of both non-hemoglobin-derived and hemoglobin-derived peptides. These findings present new grounds for further research.

Dihydroartemisinin-Piperaquine (DHAP) is a second-line antimalarial therapy for uncomplicated malaria. Abuaku et al., identified a near perfect cure rate (>90%) for DHAP, with longer prophylactic benefits over artesunate-amodiaquine and artemether lumefantrine. DHAP also improved hemoglobin levels and reduced fever in treated individuals. No evidence of DHAP resistance was reported.

Vaccine research is yet another field that cannot be ignored in the fight against malaria. Healer et al., investigated the efficacy of a protein-in-adjuvant blood stage malaria vaccine, RH5.1-CyRPA-Ripr antigen combination vaccine, relative to the single immunogen RH5 using animal models and reported low performance. The study also found the DPX^®^ platform to be the best performing formulation in potentiating *P. falciparum* inhibitory antibody responses to these antigens. Despite low performance of the vaccine, the authors encourage further exploration of other RH5 vaccine combinations.

Collectively, these articles showcase the ongoing efforts to combat malaria through diverse approaches, including the surveillance of drug resistance, developing novel antimalarial agents, evaluating combination therapies, and exploring vaccine candidates. The findings contribute to our understanding of *Plasmodium* treatment and resistance, providing a foundation for further research, and the development of innovative strategies to control malaria.

## Author contributions

LA: Writing – original draft, Writing – review & editing, Formal Analysis. EL: Writing – original draft, Writing – review & editing.

